# Risk factors and a prediction model for lower limb lymphedema following lymphadenectomy in gynecologic cancer: a hospital-based retrospective cohort study

**DOI:** 10.1186/s12905-017-0403-1

**Published:** 2017-07-25

**Authors:** Kenji Kuroda, Yasuhiro Yamamoto, Manami Yanagisawa, Akira Kawata, Naoya Akiba, Kensuke Suzuki, Kazutoshi Naritaka

**Affiliations:** Department of Obstetrics and Gynecology, Yaizu City Hospital, 1000, Dobara, Yaizu-shi, 425-8505 Japan

**Keywords:** Lower limb lymphedema, Lymph node dissection, Lymphocyst, Body mass index, Prediction model

## Abstract

**Background:**

Lower limb lymphedema (LLL) is a chronic and incapacitating condition afflicting patients who undergo lymphadenectomy for gynecologic cancer. This study aimed to identify risk factors for LLL and to develop a prediction model for its occurrence.

**Methods:**

Pelvic lymphadenectomy (PLA) with or without para-aortic lymphadenectomy (PALA) was performed on 366 patients with gynecologic malignancies at Yaizu City Hospital between April 2002 and July 2014; we retrospectively analyzed 264 eligible patients. The intervals between surgery and diagnosis of LLL were calculated; the prevalence and risk factors were evaluated using the Kaplan-Meier and Cox proportional hazards methods. We developed a prediction model with which patients were scored and classified as low-risk or high-risk.

**Results:**

The cumulative incidence of LLL was 23.1% at 1 year, 32.8% at 3 years, and 47.7% at 10 years post-surgery. LLL developed after a median 13.5 months. Using regression analysis, body mass index (BMI) ≥25 kg/m^2^ (hazard ratio [HR], 1.616; 95% confidence interval [CI], 1.030–2.535), PLA + PALA (HR, 2.323; 95% CI, 1.126–4.794), postoperative radiation therapy (HR, 2.469; 95% CI, 1.148–5.310), and lymphocyst formation (HR, 1.718; 95% CI, 1.120–2.635) were found to be independently associated with LLL; age, type of cancer, number of lymph nodes, retroperitoneal suture, chemotherapy, lymph node metastasis, herbal medicine, self-management education, or infection were not associated with LLL. The predictive score was based on the 4 associated variables; patients were classified as high-risk (scores 3–6) and low-risk (scores 0–2). LLL incidence was significantly greater in the high-risk group than in the low-risk group (HR, 2.19; 95% CI, 1.440–3.324). The cumulative incidence at 5 years was 52.1% [95% CI, 42.9–62.1%] for the high-risk group and 28.9% [95% CI, 21.1–38.7%] for the low-risk group. The area under the receiver operator characteristics curve for the prediction model was 0.631 at 1 year, 0.632 at 3 years, 0.640 at 5 years, and 0.637 at 10 years.

**Conclusion:**

BMI ≥25 kg/m^2^, PLA + PALA, lymphocyst formation, and postoperative radiation therapy are significant predictive factors for LLL. Our prediction model may be useful for identifying patients at risk of LLL following lymphadenectomy. Providing an intensive therapeutic strategy for high-risk patients may help reduce the incidence of LLL and conserve the quality of life.

## Background

Gynecologic cancers comprised 16.3% of all cancer cases in women in 2012, and were estimated to include 528,000, 320,000, and 239,000 new worldwide cases of the cervical uterus, corpus uterus, and ovaries, respectively [1]. The main treatment for gynecologic cancer is surgery, chemotherapy, and radiation therapy [2–4]. The surgical procedure involves pelvic lymphadenectomy (PLA) with or without (+/−) para-aortic lymphadenectomy (PALA), which is used for clinical staging and treatment [2–4]. PLA +/− PALA increases the volume of bleeding, duration of surgery, and postoperative complications; therefore, surgery must be evaluated and monitored properly [5, 6]. Lower limb lymphedema (LLL) is a frequent postoperative complication, and is a progressive and chronic disease characterized by lymph-carrying channel dysfunction [7] that is often accompanied by inflammation and fibrosis [8]. LLL can be asymptomatic; otherwise, symptoms include swelling, lumps, puffiness, tightness, pain, and heaviness in the leg [9]. Patients with LLL have a reduced quality of life (QOL) because of functional and cosmetic problems [10, 11]. Previous studies reported that LLL can develop any time between the immediate aftermath of surgery and many years thereafter [12–14]; hence, patients with LLL experience psychological and social burdens for extended periods of time [15].

Previous studies of risk factors for the development of LLL have been limited, and have investigated the roles of body mass index (BMI) [5, 13, 14, 16], the number of removed lymph nodes [14], the extent of lymph node dissection [9, 12], postoperative radiation therapy [14, 17], and postoperative infections [13, 14]. With respect to postoperative infections, few studies have investigated the sequence of events between the onset of infection and the subsequent onset of LLL. Moreover, studies do not always distinguish between cellulitis and lymphocyst infections. There is also scarce information on the relationship between lymphocyst formation, Chinese herbal medicines that are used by many patients, and patients’ self-management methods. Although there are several reported models for predicting lymphedema after axillary dissection in breast cancer [18–21], no prediction model for LLL has been reported to our knowledge. We therefore sought to definitively identify risk factors using retrospective statistical analysis, and to identify patients at high risk of LLL by creating a prediction model.

## Methods

**Fig. 1:**
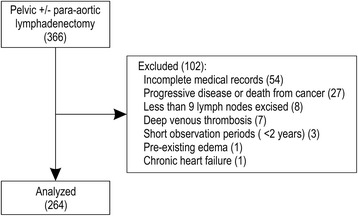
Patient enrollment flow diagram

Our study was conducted at Yaizu City Hospital in Shizuoka Prefecture, Japan. This hospital houses 471 beds and has 7 clinical oncologists; it is a major provider of gynecological and other medical care for the 400,000 people living in Yaizu and its vicinity. Based on previous studies, we calculated that a sample size of 242 patients would provide 80% power to detect the occurrence of LLL with a type I error of 5%. A total of 366 Japanese patients with gynecologic malignancies underwent PLA +/− PALA as their primary surgical treatment at the Obstetrics and Gynecology Department of Yaizu City Hospital during the period between April 1, 2002 and July 31, 2014. Informed consent regarding the therapeutic strategy was obtained from all patients before treatment. The present study was submitted and approved by the Ethics Review Committee of Yaizu City Hospital in compliance with the Helsinki Declaration. Patients’ data were collected between September 1, 2015 and November 30, 2015 from their medical records. We excluded 102 patients as shown in Fig. 1; ultimately, 264 patients were enrolled in this study.

Patients were able to seek advice over the telephone or visit the hospital for emergency consultation at any time in response to changes in symptoms, including sensations of leg heaviness, swelling, pain, fever, and erythema. We identified patients with LLL by their medical records, as documented by self-reporting as well as physical examinations performed regularly by their gynecologic oncologist, wherein both lower limbs were inspected and palpated. We also performed Doppler ultrasonography and plasma D-dimer measurements, if necessary, to rule out deep venous thrombosis. The grade of LLL was based on the most severe finding in each patient; the evaluation was conducted according to the stage scale of the International Society of Lymphology [22]. Stage 0 refers to a latent or sub-clinical condition where swelling is not yet evident despite impaired lymph transport, subtle changes in tissue fluid/composition, and changes in subjective symptoms. Stage I represents an early accumulation of fluid relatively high in protein content, which subsides with limb elevation. Stage II signifies a situation where limb elevation alone rarely reduces tissue swelling, and where pitting is manifest. Stage III encompasses lymphostatic elephantiasis where pitting can be absent and trophic skin changes such as acanthosis, further deposition of fat, fibrosis, and warty overgrowths develop. Damage to the lymphatic drainage occurs immediately in all patients who undergo lymphadenectomy, and is considered Stage 0. Because alleviation of random symptoms can occur by limb elevation, the designation of Stage I LLL may be subjective. Therefore, we considered a diagnosis of LLL to be Stage II disease or higher in order to exclude such subjective evaluation.

BMI was calculated based on a patient’s body weight immediately before surgery. While the retroperitoneal suture method was originally used for the incision between the para-aortic and pelvic areas, the areas of the internal and external iliac arteries are now left open according to procedural changes adopted in January 2013, with sutures placed only in the para-aortic area and the vaginal stump. Adjuvant therapy was then administered according to the pathological diagnosis of the extracted specimens. Postoperative radiation therapy principally involved pelvic external beam radiotherapy at 50 Gy, with the addition of vaginal brachytherapy to the vaginal stump at 12 Gy or para-aortic external beam radiotherapy at 45 Gy, as required. Adjuvant chemotherapy predominantly consisted of paclitaxel (175 mg/m^2^ once every 3 weeks or 80 mg/m^2^ once a week) and carboplatin (the area under the plasma concentration-time curve: 6.0 once every 3 weeks). The herbal medicine goshajinkigan (7.5 g/day; 2.5 g thrice daily) was administered for preventive and therapeutic purposes against paclitaxel-induced neuropathy; we tested this agent as it is also effective against edema. The presence of a lymphocyst was recorded upon detection of a cyst with a maximum diameter of ≥3 cm on ultrasonography or computed axial tomography. We investigated sites of infection (skin or lymphocyst) and estimated the onset times of LLL and infection; this determined whether LLL was actually caused by infection. Since April 2010, expert nurses have provided self-management education and guidance regarding LLL to patients who underwent lymphadenectomy. Guidance encompasses topics such as education about lymphedema mechanisms, self-measurement methods, and preventative methods against LLL that include skin care, manual lymphatic massage, compression garments, and moderate exercises.

The primary endpoint of this study was the occurrence of LLL. We calculated the durations between surgery and LLL diagnosis; patients who did not develop LLL were censored at the last follow-up date. Qualitative data were described using relative frequencies. Continuous data were expressed using the mean, standard deviation (SD), median, and interquartile range. To define the thresholds of categorical variables, we dichotomized each based on its median value: an age greater than 56 years and a number of lymph nodes greater than 42. The World Health Organization defines a normal body weight as a BMI between 18.5 to 25 kg/m^2^; therefore, a BMI of ≥25 kg/m^2^ was used as the cut-off for categorizing patients by weight. Univariate analysis of each variable was performed using the log-rank test, and the cumulative incidence was calculated using the Kaplan-Meier (KM) method. Variables were analyzed using the Cox proportional hazards method for all factors that showed *P* < 0.3 on univariate analysis, and the hazard ratios (HRs) and 95% confidence intervals (CIs) were calculated after controlling simultaneously for potential confounders. The limit of significance for the analysis was defined as a *P*-value of 0.05; 2-sided tests were used in all calculations. We determined the relative weight of each variable in the prediction model by calculating its value consistent with each coefficient of the significant variables determined by the Cox model; each coefficient was rounded to each integer for its application, and each variable was assigned a value between 0 and 6 points. To confirm the prediction model’s efficacy, we tested it in the 264 enrolled patients. Patients were classified as high-risk or low-risk based on the KM grouping for each score, and their KM curves were constructed to determine the cumulative risk of LLL. The performance of the model was evaluated using the area under the receiver operating characteristic curve (AUC). Analysis was conducted using the R statistical software (The R Foundation for Statistical Computing, Vienna, Austria, ver. 3.2.2) and EZR [23] (a modified version of R commander designed to add statistical functions frequently used in biostatistics).

## Results

Patient backgrounds along with findings and treatment methods are shown in Table 1. All 264 patients were Japanese, with a median age of 56 years (mean, 54.95; SD, 11.50; interquartile range [IQR], 46–63 years) and a median BMI of 22 kg/m^2^ (mean, 22.49; SD, 4.02; IQR, 20–25 kg/m^2^). PLA was performed in 43 patients, and PLA + PALA in 221 (up to the area above the inferior mesenteric artery in 69 patients and up to the area below the inferior mesenteric artery in 152). Lymph nodes metastasis was observed in 31 patients (11.7%). The median number of lymph node samples was 42 (mean, 42.70; SD, 18.27; IQR, 30–53). Seventeen patients with uterine cervical cancer or uterine endometrial cancer received postoperative radiation therapy. Ten patients took herbal medicine. Lymphocysts occurred in 93 patients (35.2%), unilaterally in 71 and bilaterally in 22.

**Table 1:** Clinical characteristics and treatments according to the type of cancer

	Uterine cervix	Uterine endometrium	Tube	Ovary	Vagina	*P*-value
All	78	113	2	68	3	
Age (years), Median (IQR)	47 (41–56)	60 (52–65)	65 (63–66)	56 (50–62)	58 (50–65)	<0.001
BMI (kg/m^2^), Median (IQR)	21 (19–23)	23 (29–54)	26 (25–27)	22 (19–24)	22 (21–23)	<0.001
No. of LN, Median (IQR)	39 (29–45)	43 (29–54)	53 (51–54)	44 (34–58)	31 (25–46)	0.038
Extent of lymphadenectomy						
PLA, N (%)	22 (28.2)	15 (13.3)	0(0)	5 (7.4)	1 (33.3)	0.006
PLA + PALA, N (%)	56 (71.8)	98 (86.7)	2 (100)	63 (92.6)	2 (66.7)	
LN metastasis						
Positive, N (%)	11 (14.1)	9 (8)	1 (50)	10 (14.7)	0 (0)	0.196
Negative, N (%)	67 (85.9)	104 (92)	1 (50)	58 (85.3)	3 (100)	
Retroperitoneal suture						
Yes, N (%)	58 (74.4)	82 (72.6)	2 (100)	57 (83.8)	3 (100)	0.372
No, N (%)	20 (25.6)	31 (27.4)	0 (0)	11 (16.2)	0 (0)	
Radiation therapy						
Yes, N (%)	12 (15.4)	5 (4.4)	0 (0)	0 (0)	0 (0)	0.003
No, N (%)	66 (84.6)	108 (95.6)	2 (100)	68 (100)	3 (100)	
Chemotherapy						
Yes, N (%)	53 (67.9)	67 (59.3)	2 (100)	58 (85.3)	2 (66.7)	0.002
No, N (%)	25 (32.1)	46 (40.7)	0 (0)	10 (14.7)	1 (33.0)	
Herbal medicine						
Yes, N (%)	2 (2.6)	5 (4.4)	0 (0)	3 (4.4)	0 (0)	0.872
No, N (%)	76 (97.4)	108 (95.6)	2 (100)	65 (95.6)	3 (100)	
Self-management education						
Yes, N (%)	36 (46.2)	57 (50.4)	1 (50)	26 (38.2)	0 (0)	0.266
No, N (%)	42 (53.8)	56 (49.6)	1 (50)	42 (61.8)	3 (100)	
Infection						
Skin (cellulitis), N (%)	4 (5.1)	9 (8.0)	0 (0)	3 (4.4)	0 (0)	0.809
Lymphocyst, N (%)	3 (3.8)	6 (5.3)	0 (0)	6 (8.8)	0 (0)	
No, N (%)	71 (91.0)	98 (86.7)	2 (100)	59 (86.8)	3 (100)	
Lymphocyst formation						
Yes, N (%)	23 (29.5)	37 (32.7)	1 (50)	32 (47.1)	0 (0)	0.095
No, N (%)	55 (70.5)	76 (67.3)	1 (50)	36 (52.9)	3 (100)	
No. of patients excluded owing to their medical history						
Before surgery	0	0	1	2	0	
After surgery	0	4	0	2	0	

Definition of *abbreviations*: *IQR* Interquartile range, *BMI* Body mass index, *No.* Number, *LN* Lymph node, *PLA* Pelvic lymphadenectomy, *PALA* Para-aortic lymphadenectomy

**Fig. 2:**
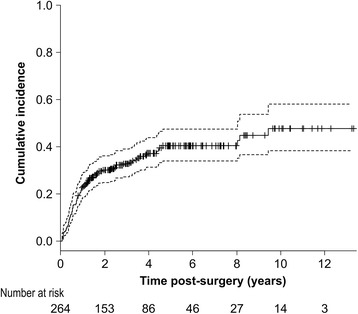
Kaplan-Meier plot of the cumulative incidence of lower limb lymphedema

The median follow-up duration for the 264 patients was 2064 days (range: 365–4868 days), with LLL occurring in 97 patients during this period. Figure 2 shows the cumulative incidence using the KM method. The cumulative incidence rate of LLL at 10 years was 47.7% (95% CI, 36.9–56.7%). The incidence reached 50% for those in which the condition occurred by 13.5 months. The cumulative incidences for each post-surgical year are shown in Table 2. Based on the cumulative number of patients at 10 years after surgery, the cumulative percentages were 48.4% within the first year, 63.3% within the second year, 68.8% within the third year, and 84.7% within the fifth year.

**Table 2:** Cumulative incidence by Kaplan-Meier

Years after surgery (y)	Cumulative incidence (Wy)	Std Er	95% CI	Ratio (^a^)
1	0.231	0.0078	0.179–0.281	0.484
2	0.302	0.0124	0.243–0.356	0.633
3	0.328	0.0145	0.266–0.384	0.688
5	0.404	0.0233	0.333–0.468	0.847
8	0.426	0.0293	0.343–0.498	0.893
10	0.477	0.0458	0.369–0.567	1.000

Definition of *abbreviations*: *Std Er* Standard error, *CI* Confidence interval

^a^Cumulative ratio for each year on the basis of the number of LLL patients within 10 years after surgery: Wy/W10

**Fig. 3:**
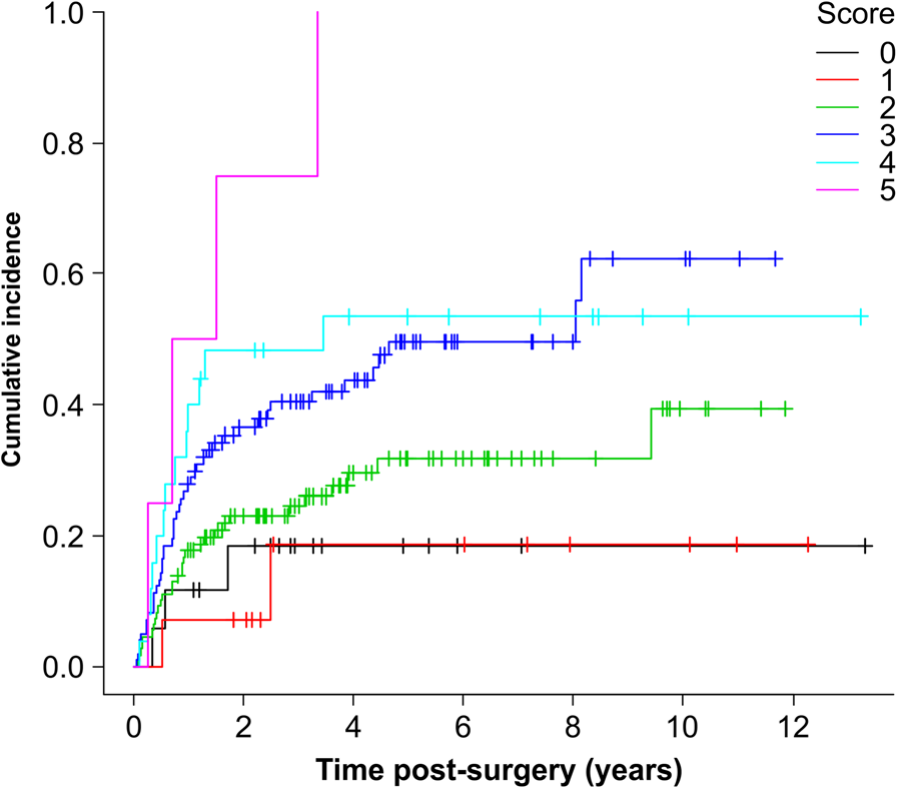
Cumulative incidence of lower limb lymphedema according to the predictive score

**Fig. 4:**
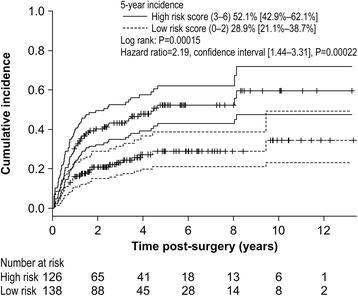
Cumulative incidence of lower limb lymphedema according in the low- vs. high-risk groups

Table 3 shows the results of univariate analysis (log-rank test) for each lymphedema risk factor. Variables extracted by univariate analysis were entered into the Cox model, and the results are displayed in Table 4. The factors shown to have a significant influence on LLL were BMI ≥25 kg/m^2^, PLA + PALA, radiotherapy, and lymphocyst formation. However, LLL did not correlate with age, type of cancer, number of lymph nodes, retroperitoneal suture, chemotherapy, lymph node metastasis, herbal medicine, self-management education, or infection. We assigned each variable a value according to its significance as determined by the Cox model as follows: BMI ≥25 kg/m^2^: 1 point, PLA + PALA: 2 points, radiotherapy: 2 points, and lymphocyst formation: 1 point. All 264 patients were scored (Fig. 3) and stratified into a low-risk group (those with cumulative scores of 0–2 points; 138 patients) and a high-risk group (those with cumulative scores of 3–6 points; 126 patients). Figure 4 compares the cumulative incidence of each group; the incidence of LLL in the high-risk group was significantly higher compared to that in the low-risk group. The cumulative incidence at 5 years was 52.1% [42.9%–62.1%] for the high-risk group and 28.9% [21.1%–38.7%] for the low-risk group. The AUC for the prediction model was 0.631 at 1 year, 0.632 at 3 years, 0.640 at 5 years, and 0.637 at 10 years.

**Table 3:** Incidences of LLL according to risk factors

Variables	Variable category	No. of patients	No. of patients with LLL (%)	*P*-value
Age, (years)	<56	127	40 (31.5)	0.089
	≥56	137	57 (41.6)	
BMI, (kg/m^2^)	<25	194	66 (34.0)	0.145
	≥25	70	31 (44.3)	
Type of cancer	Cervix	78	26 (33.3)	0.569
	Body	113	46 (40.7)	
	Tube	2	1 (50.0)	
	Ovary	68	24 (35.3)	
	Vagina	3	0 (0)	
Extent of Lymphadenectomy	PLA	43	10 (23.3)	0.045
	PLA + PALA	221	87 (39.4)	
No. of LN	<42	128	43 (33.6)	0.202
	≥42	136	54 (39.7)	
LN metastasis	Yes	31	9 (29.0)	0.270
	No	233	88 (37.8)	
Retroperitoneal suture	Yes	202	78 (38.6)	0.609
	No	62	19 (30.6)	
Radiation therapy	Yes	17	9 (52.9)	0.298
	No	247	88 (35.6)	
Chemotherapy	Yes	182	65 (35.7)	0.423
	No	82	32 (39.0)	
Herbal medicine	Yes	10	6 (60.0)	0.108
	No	254	91 (35.8)	
Self-management education	Yes	120	38 (31.7)	0.724
	No	144	59 (41.0)	
Infection	Skin (cellulitis)	9	6 (66.7)	0.105
	Lymphocyst	11	6 (54.5)	
	No	244	85 (34.8)	
Lymphocyst formation	Yes	93	46 (49.5)	0.003
	No	171	51 (29.8)	

Definition of *abbreviations*: *IQR* Interquartile range, *BMI* Body mass index, *No.* Number, *LLL* Lower limb lymphedema, *LN* Lymph node, *PLA* Pelvic lymphadenectomy, *PALA* Para-aortic lymphadenectomy

**Table 4:** Cox hazard analysis of LLL risk factors

	Coefficient	Hazard ratio	95% CI	*P*-value
Age [≥56 years]	0.302	1.353	0.892–2.052	0.154
BMI [≥25 kg/m^2^]	0.480	1.616	1.030–2.535	0.037
PLA + PALA	0.843	2.323	1.126–4.794	0.023
No. of LN [≥42]	−0.022	0.978	0.631–1.515	0.920
LN metastasis	−0.481	0.618	0.305–1.251	0.181
Radiation therapy	0.903	2.469	1.148–5.310	0.021
Herbal medicine	0.613	1.846	0.779–4.376	0.164
Inf [skin (cellulitis)]	0.848	2.334	0.984–5.535	0.054
Inf [lymphocyst]	0.176	1.192	0.496–2.867	0.695
lymphocyst formation	0.541	1.718	1.120–2.635	0.013

Definition of *abbreviations*: *BMI* Body mass index, *CI* Confidence interval, *Inf* infection, *PLA* Pelvic lymphadenectomy, *LLL* Lower limb lymphedema, *LN* Lymph node, *No.* Number, *PALA* Para-aortic lymphadenectomy

## Discussion

In this study, we determined the risk factors for LLL to be BMI ≥25 kg/m^2^, PLA + PALA, lymphocyst formation, and postoperative radiation therapy. The effects of self-management education and Chinese herbal medicines, for which previous reports are scarce, were not found to be associated with LLL. Postoperative lower limb cellulitis and infected pelvic cavity lymphocysts did not significantly induce LLL. We also found that our new prediction model constructed according to the 4 risk factors reliably classified patients into high-risk and low-risk groups.

A positive association has been established between BMI and upper limb lymphedema in breast cancer [24, 25]. Previous studies of LLL, however, showed that its development may be associated with a higher BMI [26], a lower BMI [16], or have no association at all [5, 13]. We explored this aspect using the BMI as measured immediately before surgery, and divided the patients into 2 categories using BMI ≥25 kg/m^2^ as a cutoff for “overweight” or “obese” patients. Accordingly, we showed that a higher BMI was associated with the development of LLL. As the amount of adipose tissue increases in the lower extremity, lymphatic vessels may become dysfunctional, thereby reducing proximal lymphatic flow [26]. It has been suggested that obesity increases perioperative complications [27–29]; therefore, it will be necessary to perform proper weight management once surgery is planned.

Regarding the extent of lymphadenectomy, we compared PLA + PALA to PLA alone. While no previous study has shown a significant association [9], we found that patients who underwent PLA + PALA have a higher risk of LLL. Recent studies revealed the dissection of circumflex iliac lymph nodes (CILs) to be an important risk factor [13, 30], although CILs were removed for all patients in our study. These results suggest that CILs have a greater impact on the development of LLL than PALA. CILs are located between the deep circumflex iliac vein and the femoral canal, and are involved in draining the lymph nodes of the lower limbs in the pelvis [13, 31]. Therefore, CILs ought to be preserved for patients at lower-risk of CIL metastasis [12, 13, 31]. Positron emission tomography-computed tomography and sentinel lymph node biopsy may be investigated further in order to determine an appropriate lymph node dissection range [5, 32, 33].

Our study showed a significant positive correlation between postoperative radiation therapy and LLL. Although radiation therapy was not a risk factor for LLL [14], studies have shown a significantly higher incidence of LLL in patients who underwent this type of therapy [17, 34]. Radiation therapy may prevent lymphatic reconstruction by inducing tissue fibrosis in the irradiated area [35]. As chemotherapy may be a substitute for postoperative irradiation under certain conditions, its adaptation should be examined in future studies [36, 37].

We found a positive correlation between lymphocyst formation and the development of LLL, which is consistent with the findings of a previous study [14]. These results suggest that the damage to the lymphatic system by surgery prevents the flow of lymphatic vessels; hence, lymphocyst formation may result from incomplete collateral lymphatic circulation. However, other reports indicated no significant correlation [12, 13], and this discrepancy may be due to the differences in the sizes, numbers, and symptoms of lymphocysts, as well as the imaging methods used to evaluate them. The development of future surgical techniques should therefore focus on suppressing the onset of lymphocysts.

Inflammation activates fibroblasts and causes hyperplasia of collagen fibers, which leads to lymphedema development [8]. Previous reports have suggested a correlation between cellulitis and LLL [13, 14]; in order to confirm whether LLL was caused following cellulitis and lymphocyst infection, the times of LLL and infection onset were investigated in particular detail. In the present study, 31 patients with postoperative infections included 16 with lower limb cellulitis and 15 with lymphocyst infection. Of these, 20 patients developed LLL following infection with a median period to onset of 414 days (IQR, 63–1274 days), indicating no significant correlation between infection and the development of LLL. However, the analysis of 31 patients in whom infection occurred before and after LLL (Table 5) indicated a strong positive correlation (HR, 4.923; 95% CI, 2.585–9.381). Hence, the possibility should be considered that there is an interval between symptom onset and diagnosis of infection or LLL.

**Table 5:** Clinical data of 31 patients in whom infection occurred before and after LLL

Onset process	Infection → LLL	LLL → Infection
	*n* = 20	*n* = 11
Onset periods*		
< 1 year	9	8
≥ 1 to <2 years	4	1
≥ 2 years	7	2
Type of infection		
Cellulitis	9	7
Lymphocyst infection	11	4

*Periods diagnosed with LLL after infection or diagnosed with infection after LLL

LLL = lower limb lymphedema

Until December 2012, we sutured the entire retroperitoneum at the last stage of lymphadenectomy. In January 2013, however, we began to constrain suturing only to the vaginal stump and para-aortic areas, and did not perform suturing in the external and internal iliac vessel area while using an adhesion-reducing agent instead. However, no significant difference between the 2 methods was observed in this study. Several reports documented similar findings [12, 13], although another suggested that non-closure in the retroperitoneum reduced lymphocyst formation and LLL [38]. Leaving the retroperitoneum open may decrease lymphocyst formation, reducing lymphedema as a result.

Chinese herbal medicines that are reportedly used to treat lymphedema include goreisan, saireito, and goshajinkigan [39, 40]. These agents possess diuretic or anti-inflammatory properties, although their mechanisms and effects have not been fully elucidated. We investigated patients who took goshajinkigan for neuropathy, but were unable to confirm its efficacy. It may be necessary to investigate herbal medicines in a larger population to determine their true effects.

We provided self-management education on LLL to patients after lymphadenectomy starting in April 2010; however, we found no significant correlation between self-management education and the development of LLL in our study. Skin care during self-management is generally not useful on its own, but is one component of composite therapy [41]. In upper lymphedema, manual (simple) lymph drainage had a preventive effect [42], and a program of slowly progressive weight lifting did not result in an increased incidence of lymphedema in the arms despite guidance advising breast cancer survivors to avoid lifting children, heavy bags, or other weighty objects [43]. In contrast, similar investigations have not yet been performed for the lower limbs. The reason may be that the lower limbs are more likely to be impacted by gravity than the upper limbs because of postoperative lifestyles or employment conditions [44]. We did not investigate how each patient currently practiced self-management at home; therefore, we advocate for advanced physical therapy to prevent LLL development.

Our data indicated that the incidence rate of LLL increases logarithmically; this rate was 32.8% at 3 years and 47.7% at 10 years. Previous studies have revealed incidences of LLL ranging from 1.2% to 58% [5, 14, 45–47]; however, these studies had varying observation periods, diagnostic criteria, patient backgrounds, and other such parameters. In particular, we graded LLL based on the most severe findings on both the lower limbs; however, the evaluation methods vary in different studies (particularly the diagnostic criteria). More importantly, we focused not on the differences in incidence rates between institutions but on the identification of causative and predictive factors for LLL. The prediction model derived from our data is useful to clinicians for identifying patients’ postoperative statuses, including staging, tissue types, complications, and other factors, and for decision-making regarding treatment. Patients who undergo lymph node dissection will be able to consider treatment options, including adjuvant therapy, depending on the calculated risk before surgery or even during postoperative follow-up, based on our model. Although a mainstay therapy for LLL has not yet been established, intensively therapeutic strategies for high-risk patients would prevent or reduce incidences of LLL and therefore improve the QOL of patients following lymphadenectomy. These retrospective data lay the groundwork for future prospective studies; at the same time, a larger retrospective study based on multicenter databases ought to be performed in order to improve the accuracy of our prediction model.

The limitations of our study include its retrospective nature, the fact that its data are derived from a single center with all Japanese patients, its limited sample size, and diagnostic bias. With respect to the latter, early stage subclinical lymphostasis (microlymphedema) is often not apparent on physical examination immediately after surgery. In recent years, lymphedema has also been evaluated using perometry and bioimpedance [48–51]. LLL might be diagnosed accurately if patients were evaluated using such devices during follow-up; however, these devices are not yet widely available because of their high cost. Therefore, the development of improved diagnostic criteria and methods of measurement is an important future goal. Additionally, we did not compare physical and psychological QOL changes using numerical/quantitative scales before vs. after the occurrence of lymphedema, it is worth investigating the correlation between the degree of lymphedema and QOL in each patient in the future.

## Conclusion

We found that a BMI ≥25 kg/m^2^, PLA + PALA, lymphocyst formation, and postoperative radiation therapy are independent predictive factors for the development of LLL. A new prediction model constructed using these 4 factors was able to classify patients into high-risk and low-risk groups for LLL development. This model may be useful for predicting LLL in patients following lymphadenectomy, thus permitting intensive therapeutic strategies for high-risk patients aimed at reducing the risk of LLL development and conserving the QOL.

## Abbreviations

AUC: Area under the receiver operating characteristic curve
BMI: Body mass index
CI: Confidence interval
CIL: Circumflex iliac lymph node
HR: Hazard ratio
IQR: Interquartile range
KM: Kaplan-Meier
LLL: Lower limb lymphedema
PALA: Para-aortic lymphadenectomy
PLA: Pelvic lymphadenectomy
QOL: Quality of life
SD: Standard deviation
